# The Florida Mobile Health Adherence Project for People Living With HIV (FL-mAPP): Longitudinal Assessment of Feasibility, Acceptability, and Clinical Outcomes

**DOI:** 10.2196/14557

**Published:** 2020-01-08

**Authors:** César Escobar-Viera, Zhi Zhou, Jamie P Morano, Robert Lucero, Spencer Lieb, Sean McIntosh, Kevin A Clauson, Robert L Cook

**Affiliations:** 1 Center for Research on Media, Technology, and Health University of Pittsburgh Pittsburgh, PA United States; 2 College of Public Health and Health Professions University of Florida Gainesville, FL United States; 3 Division of Infectious Disease and International Medicine Morsani College of Medicine University of South Florida Tampa, FL United States; 4 College of Nursing University of Florida Gainesville, FL United States; 5 The AIDS Institute Tampa, FL United States; 6 College of Pharmacy & Health Sciences Lipscomb University Nashville, TN United States; 7 Southern HIV and Alcohol Research Consortium Center for Translational HIV Research University of Florida Gainesville, FL United States

**Keywords:** mHealth, HIV, ART adherence, feasibility, acceptability

## Abstract

**Background:**

For people living with HIV (PLWH), antiretroviral therapy (ART) adherence is crucial to attain better health outcomes. Although research has leveraged consumer health information technologies to enhance ART adherence, no study has evaluated feasibility and clinical outcomes associated with the usage of a commercially available, regularly updated mobile health (mHealth) app for improving ART adherence among PLWH.

**Objective:**

This study aimed to assess the feasibility, acceptability, and clinical outcomes of Care4Today, an existing, free, biprogrammatic mHealth app for improving ART adherence among PLWH.

**Methods:**

The Florida mHealth Application Adherence Project (FL-mAPP) was a 90-day longitudinal pilot study conducted in 3 public HIV clinics in Florida, United States. After obtaining informed consent, 132 participants completed a survey and then were given the option to try an existing mHealth app to help with ART adherence. Of these, 33.3% (44/132) declined, 31.1% (41/132) agreed but never used the app, and 35.6% (47/132) used the app. All were asked to complete follow-up surveys at 30 days and 90 days after enrollment. Usage data were used to assess feasibility. Clinical outcomes of self-reported ART adherence and chart-obtained HIV viral load and CD4+ T-cell counts were compared among those who used the platform (users) versus those who did not (nonusers). Participants and HIV care providers also provided responses to open-ended questions about what they liked and did not like about the app; comments were analyzed using thematic analysis.

**Results:**

Of 132 participants, 47 (35.6%) and 85 (64.4%) were categorized as users and nonusers, respectively. Among users, a Kaplan-Meier plot showed that 25 persons (53%) continued using the app after the 90-day follow-up. At 30-day follow-up, 13 (81.3%) of those who used the mHealth app reported ≥95% ART adherence, compared with 17 (58.6%) nonusers (*P*=.12). Overall, 39 (82%) users liked or somewhat liked using the platform. Participants’ favorite features were medication reminders, ability to create custom reminders, and adherence reports.

**Conclusions:**

This longitudinal study found that a commercially available medication adherence mHealth app was a feasible and acceptable intervention to improve ART adherence among PLWH and engaged in clinical care across 3 public HIV clinics in the state of Florida. Overall, participants liked the Care4Today app and thought the medication reminders were their favorite feature. Generally, self-reports of ART adherence were better among users than nonusers, both at 30- and 90-day follow-ups. Further clinical research needs to address user fatigue for improving app usage.

## Introduction

### Background

For people living with HIV (PLWH), antiretroviral therapy (ART) adherence is crucial to attain both viral load suppression and optimal health outcomes. Although up to 85% of PLWH in the United States repor*t* >95% ART adherence [1], concerns remain about the dynamic nature of and potential decline in adherence over time [2-10]. Although research has leveraged consumer health information technologies to enhance ART adherence, such as SMS [11-25] and mobile health (mHealth) apps [26-31], no study has evaluated the longitudinal feasibility and acceptability of an existing, commercially available, free, and regularly updated mHealth app for improving ART adherence among PLWH [32].

Although 3 meta-analyses [33-35] found SMS reminders to be efficacious for improving ART adherence across diverse populations, the effects were modest and declined over time [34]. Given most studies were descriptive, evidence for mHealth apps that improve HIV medication adherence is limited [36,37]. For example, previous research showed mobile apps to improve ART adherence are feasible and acceptable [28,38], and reminders seem to be effective in helping initiate a medication adherence routine. Data-guided counseling may help PLWH to identify strategies to overcome adherence barriers [39]. However, mHealth interventions seem to have a limited scope during the efficacy trial phase, failing to incorporate functions identified as useful by PLWH [40]. In addition, the traditional research approach of developing and testing homegrown mHealth apps may no longer be practical, given the rapid pace of innovation in mHealth technologies and the budgetary requirements of maintaining, debugging, updating, and upgrading a mobile app [41,42].

Care4Today Mobile Health Manager (Johnson & Johnson) is a commercially available mHealth app designed to improve medication adherence and has been described in detail elsewhere [43]. The app is biprogrammatic (ie, designed for both smartphones [iPhone or Android] and SMS interfaces). Previously, we reported that among PLWH recruited from public HIV clinics in Florida, United States, over 70% of participants were interested in using an app to monitor their medication adherence. We found that perceived need and availability of someone at the clinic to assist with the app were strongly associated with willingness to try it [43]. These findings informed the implementation of the study we report here.

### Objectives

The objective of this study was to assess the feasibility and acceptability of Care4Today among PLWH. More specifically, we sought to evaluate (1) app usage pattern; (2) changes in self-reported ART adherence, HIV viral load, and CD4+ T-cell count among PLWH who used the app compared with those who did not; and (3) preferences and suggestions for app features for PLWH. As a secondary goal, we sought to assess the interest of HIV providers and clinical staff in receiving app-generated adherence data.

## Methods

### Design, Participants, and Setting

The Florida mHealth Application Adherence Project (FL-mAPP) was a 90-day feasibility and acceptability longitudinal pilot study of a commercially available mHealth app for treatment adherence among PLWH, conducted from 2015 to 2016. As previously described, participants were recruited from public HIV clinics (2 urban and 1 rural) affiliated with the Florida Department of Health [43]. Eligibility criteria included (1) having an HIV positive laboratory result and having been prescribed ART, (2) documented viral load or scheduled viral load test within 4 weeks before or after enrollment, (3) 18 years of age or older, (4) capable of reading and speaking English, (5) owner of a smartphone or feature phone, and (6) able and willing to incur the cost of receiving and sending SMS messages. Potential participants were excluded if they were visually impaired or if they shared their phone with another person.

After providing informed consent, participants completed a survey questionnaire. They were then able to choose by trying the app in 2 forms: (1) SMS reminders only (for flip phone owners) or (2) full app (for smartphone owners). Clinic personnel assisted participants as needed with downloading the app, creating their account, and configuring the app or SMS reminders. All participants were asked to complete 2 follow-up surveys at 30 and 90 days after enrollment. Participants who chose to try the app or SMS reminders were asked if we could submit a dashboard-generated adherence report to be reviewed by their provider as part of ongoing clinical care. All of these participants accepted the request.

Participants received gift cards totaling US $30 over the course of the study in compensation for their time completing the surveys regardless of the decision to use the app. Follow-up clinical measures included 30- and 90-day self-reported ART adherence, HIV viral load, and CD4+ T-cell count.

### Data and Measures

Surveys were conducted at baseline, interim (day 30), and endpoint (day 90). Baseline paper surveys were completed in person at the clinics, and follow-ups were conducted either in person or over the phone by research staff, as described previously [43]. The baseline survey assessed demographic characteristics, self-reported ART adherence, and mobile phone and mHealth app use. Interim and endpoint surveys assessed self-reported ART adherence only.

#### Demographic Variables

Participants reported their age in years (categorized in 4 groups [ie, 18-29, 30-35, 37-39, 43-49, and 50 and older]), gender assigned at birth, race and ethnicity (categorized as Hispanic; white, non-Hispanic; black, non-Hispanic; and other, non-Hispanic), highest education level attained (less than high school, high school graduate or general education diploma, and more than high school), and marital status (single/never married, divorced/widowed/separated, and married/living with a long-time partner).

#### Feasibility

We assessed usage of the app at 30 and 90 days after enrollment among those who used the SMS or app at least once. For each participant, continued use was defined as number of days elapsed from enrollment until the last time the participant reported either a taken or a missed dose via the app or SMS. For analysis, persons who declined to try the app and those who agreed to try but never actually used it were categorized together as *nonusers*, and those who used the app at least once were defined as *users*.

#### Clinical Outcomes: Antiretroviral Therapy Adherence, HIV Viral Load, and CD4+ T-Cell Count

We measured self-reported ART adherence using a previously developed item that was validated for PLWH [44]. Participants were asked, *Over the last 30 days, how many days did you forget or miss your HIV medications, even partially?* We categorized participants’ ≥95% adherence if they reported missing doses on less than 2 days in the previous month. We also attempted to define ART adherence according to the responses from the app, but if participants did not report on a specific day, it was not possible to tell if they were nonadherent or just forgot to enter information into the system.

We collected HIV viral load and CD4+ T-cell count at 2 distinct intervals: (1) baseline viral load and CD4+ T-cell count at enrollment day or the 30 days preceding enrollment and (2) the next available medical record laboratory results after enrollment. We used a plasma level cut-off point of less than 40 copies/ml of HIV-1 RNA to define optimal viral suppression.

#### Acceptability

At study day 30, participants who chose to try the app answered open-ended questions that assessed acceptability [45]. Items included the following: *How did you like using the Care4Today system? What stopped you from using the system more? Which features did you like the most? What did you not like about Care4Today? How can the system be improved?*

#### Provider Survey

Participant providers were invited to participate in the study by receiving printed copies of app-generated adherence reports at day 30, 60, and 90. After day 90 of the study, consenting providers completed brief surveys regarding their interest in receiving the app-generated adherence data. Items included the following: *How interested would you be in continuing to receive information on patients’ self-reported adherence with this dashboard?* *How often would you like to see these type of data about your patients?* and *How interested would you be in learning how to access the dashboard online to look at adherence reports of your patients who are using the “Care4Today” app?*

### Data Analyses

For quantitative data, proportions and chi-square tests compared baseline demographic characteristics, ART adherence, and clinical variables between users versus nonusers [43], both at baseline and follow-up. A Kaplan-Meier plot curve described the proportion of persons who continued to use the app over time. To measure ART adherence, we compared ≥95% versus <95% ART adherence at follow-up among users and nonusers with poor adherence at baseline (n=57). Then, chi-square tests were used to determine the difference in adherence between those who used the mHealth platform and those who did not, at days 30 and 90 after enrollment.

For responses to open-ended questions, we used an inductive thematic analysis [46] to unveil barriers and facilitators of app use. Participant answers were organized in 2 separate spreadsheets. In total, 2 of the authors (RLC and CGEV) created initial codes and labeled the data using these codes. Next, the codes were categorized into themes and interpreted. Throughout the process, discrepancies were resolved among the qualitative research team.

### Ethics and Consent Process

All participants were provided with written informed consent, and Health Insurance Portability and Accountability Act required health authorization to use personal health information for research. The institutional review boards of the Florida Department of Health, the University of South Florida, and the University of Florida reviewed and approved all procedures and data management processes.

## Results

### Participant Characteristics and Mobile Health Platform Feasibility

**Figure 1:**
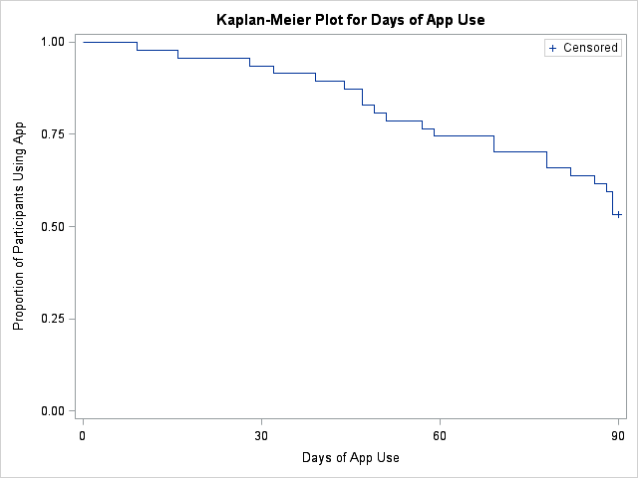
Proportion of persons who continued to use the app to input antiretroviral therapy adherence information after enrollment (N=47).

As previously reported by this study group [43], of the total 132 participants at baseline, 47 (47/132, 35.6%) agreed to use the app and used it at least once (*users*). *Nonusers* (85/132, 64.4%) comprised 44 participants (44/132, 33.3%) who declined to try the app and 41 (41/132, 31.1%) who agreed to try the app but did not use it. Of 47 *Users*, 37 (78%) who chose to use the full smartphone app and 10 (21%) who chose SMS. We conducted an analysis to compare these 2 groups and found no significant differences other than smartphone ownership, which was significantly more frequent among those who chose to use the full app. At baseline and follow-up, no significant differences were found in ART adherence, HIV viral load, or CD4+ T-cell count between users and nonusers (Table 1). Among users, more than 50% continued to use the app for 90 days or more, with 6% (3/47) using the app for less than 30 days, 19% (9/47) between 30 and 59 days, 21% (10/47) between 60 and 89 days, and 53% (25/47) for over 90 days (Figure 1).

From baseline, 81% (38/47) of app users and 64% (54/85) of nonusers completed 90-day follow-up surveys. Table 2 shows the proportion of persons who reported >95% adherence at 30- and 90-day follow-up among participants that reported <95% ART adherence at baseline. At enrollment, 57/132 persons (44.5%) reported <95% ART adherence. At 30-day follow-up, we found a nonsignificant difference (*P*=.12) between ≥95% adherence reported by app users (81%, 13/16) compared with just over half of nonusers (58%, 17/29). Similarly, at 90-day follow-up, 7/15 users (46%) reported ≥95% ART adherence compared with 9/22 nonusers (40%), although this difference was not significant.

**Table 1:** Baseline and follow-up of antiretroviral adherence and clinical parameters of Care4Today users and nonusers, Florida Mobile Health Application Adherence Project study, 2015 to 2016 (N=132).

Characteristics		Total (N=132)^a^, n (%)	Users (n=47), n (%)	Nonusers (n=85), n (%)	*P* value^b^
**Antiretroviral therapy adherence ≥95%**					
	Baseline	71 (55.5)	26 (57.8)	45 (54.2)	0.7
	30-day follow-up	85 (79.4)	35 (85.4)	50 (75.8)	0.23
	90-day follow-up	65 (70.7)	25 (65.8)	40 (74.1)	0.39
**HIV RNA ≤200 copies/mL**					
	Baseline	91 (71.0)	37 (78.7)	54 (66.7)	0.15
	90-day follow-up	97 (88.2)	60 (85.7)	37 (92.5)	0.37
**CD4 coun*t* >500 cells/µL**					
	Baseline	69 (54.3)	25 (54.4)	44 (54.3)	0.99
	90-day follow-up	61 (56.0)	23 (59.0)	38 (54.3)	0.64
**Mean CD4 count (cells/µL), mean (SD)**					
	Baseline	575 (311)	560 (329)	583 (280)	0.68
	90-day follow-up	629 (346)	635 (352)	626 (345)	0.88

^a^Some variables might not total 132 owing to missing data.

^b^*P* value derived using chi-square analysis comparing proportions in each category.

**Table 2:** Self-reported antiretroviral adherence and clinical parameters at 30- and 90-day follow-up among participants with <95% adherence at baseline (N=57).

Parameter			Users (n=19)^a^	Nonusers (n=38)^b^	*P* value^c^
**Self-reported antiretroviral therapy adherence, n (%)**					
	**30-day follow-up**				.12
		≥95%	13 (81)	17 (58)	
	**90-day follow-up**				.73
		≥95%	7 (46)	9 (40)	
**HIV viral load** **, n (%)**					
	**Baseline**				.30
		<40 copies/mL	12 (63)	17 (48)	
	**Follow-up**				.74
		<40 copies/mL	12 (75)	20 (69)	
**CD4+ T-cell count** **, n (%)**					
	**Baseline**				.13
		0-200 cells/mL^3^	5 (26)	2 (5)	
		201-500 cells/mL^3^	6 (31)	14 (40)	
		>500 cells/mL^3^	8 (42)	19 (54)	
	**Follow-up**				.36
		0-200 cells/mL^3^	4 (26)	3 (10)	
		201-500 cells/mL^3^	5 (33)	10 (34)	
		>500 cells/mL^3^	6 (40)	16 (55)	

^a^Some variables might not be total 19 owing to missing data.

^b^Some variables might not be total 38 owing to missing data.

^c^*P* value derived using chi-square analysis comparing proportions in each category.

### Acceptability

Overall, at 30-day follow-up, there were 47 users who used the app at least once, of whom 39/47 (82%) liked or somewhat liked using the app, 1/47 (2%) did not like it, and 7/47 (15%) did not respond the question. Favorite features were medication reminders (n=25), ability to create custom reminders (n=5), adherence reports (n=5), and multiple features (n=4). Open-ended questions identified app features and technical issues that participants found challenging. The qualitative findings are available in Multimedia Appendix 1. Users reported that these issues kept them from using the app and could be improved for future use. Among these, lack of consistency in receiving reminders, characteristics of these reminders (eg, frequency, sound type, and duration), and inability to use all the features when not connected to the internet were main complaints and barriers to use. In terms of the features, users wanted more flexibility to report their medication taking, both if medication was taken a little early or a little late. Suggestions for improvement focused on enhancing the reminders in frequency, recurrence, or volume. Nevertheless, 36/47 users (80%) reported that they intended to keep using the Care4Today app.

### Provider Survey

A total of 10 clinicians completed the survey: 5 nurse practioners and 5 physicians. The average age was 47.8 years (SD 15.6) with the majority being female (80%, 8/10) and white non-Hispanic (70%, 7/10). Of the 10 providers, 6 had over 10 years of experience working with PLWH, 1 had 6 to 10 years, and 3 had 5 years or less. Although 90% (9/10) of providers were either very or somewhat interested in reviewing adherence reports through the app, 80% (8/10) wanted to see these reports either monthly or quarterly (as opposed to daily or weekly). Finally, 5 providers (3 nurse practitioners and 2 physicians) had low to no interest in logging in to the provider interface to access the reports.

## Discussion

### Principal Findings

This longitudinal study found that a commercially available medication adherence mHealth app was a feasible and acceptable intervention to improve ART adherence among PLWH engaged in clinical care across 3 public HIV clinics in the state of Florida. To our knowledge, this is the first feasibility pilot study that used a free, commercially available mHealth app to improve medication adherence among PLWH. Overall, participants liked the Care4Today app and favored the medication reminders feature. Generally, self-reports of ART adherence were better among users than nonusers, both at 30- and 90-day follow-ups, although the difference between groups was not statistically significant. Given the main goal of this study was to assess acceptability and feasibility of the intervention, we did not intend to demonstrate statistical significance of our findings. Rather, we sought to identify trends and factors that might inform a larger efficacy study. Importantly, although our findings do not support larger implementation of commercially available medication adherence apps for PLWH at this time, we did identify promising results in clinical outcomes that are commonly accepted proxies for efficacy (ie, self-reported ART adherence).

Among participants who had less than optimal ART adherence at baseline, over half of them started and kept using the intervention for the entire duration of the study. Although the drop-in usage between 30- and 90-day follow-ups might seem considerable when compared with previous research [30,47], some clarifications might help to interpret these findings. On one hand, study participants were not financially compensated to use the platform, and all usage was completely voluntary. On the other hand, almost half of the platform users already reported an ART adherence of ≥95% at baseline; therefore, some participants might have found the intervention less critical for their compliance than others. It would be appropriate to have future research on commercially available adherence apps looking more specifically at usage behavior of a larger group of persons with less than optimal ART adherence at the start of the study.

Of our participants with less than optimal ART adherence at baseline, the proportion of persons who reported good ART adherence at follow-up was higher among app users than nonusers. Although this difference was not statistically significant, this finding suggests that use of the app did improve medication taking, and this could be assessed with a larger clinical trial. Although self-reported ART adherence has been consistently associated with improved clinical outcomes, including viral suppression [48,49], most self-report measures are a proxy of true adherence and present some limitations. To address this limitation, we used a carefully validated measure of self-report ART adherence that is both simple and convenient for PLWH [44].

Participants’ feedback focused on suggestions to improve features and technical aspects of the app. Among these, suggestions about personalized and customized reminder characteristics were paramount. Moreover, users wanted an app that would work even if they were off a wireless internet connection. These recommendations speak about the user’s desire of more independence when using mHealth apps. However, these requests need to be balanced with the need to keep apps efficient and fun to use. Potentially, these objectives can be met with commercially available apps that are developed and maintained by dedicated personnel in charge of updating and enhancing app databases and features. On the contrary, providers’ feedback showed clinicians see value in accessing self-reported adherence data from their patients during clinical visits. This seems to fit well with the clinical workflow; visits provide the opportunity to address adherence inconsistencies that may be reported through the mHealth app reports. However, in order for providers to retrieve these data on their own, mHealth apps should be able to easily communicate with information systems already being used by health professionals. Clinicians had less interest in viewing these reports on a daily or weekly basis. One potential way to link these reports into a workflow could be to establish a critical number of missing doses that would trigger an increased frequency of medication reminders, or suggestions for tele-consultations with clinical staff in advance of the next visit to address adherence inconsistencies promptly.

### Limitations and Summary

Our work has several limitations. This study was conducted at 3 HIV clinics affiliated to the Florida Health Department. Thus, our findings cannot be generalized to sites that operate with different procedures for patient engagement and follow-up. However, our work is of interest given that these clinics draw from a distinct socioeconomic group of diverse backgrounds. Almost two-thirds of study participants were categorized as app nonusers, either because they declined to try the app at all, had problems downloading and using the app, or did not use it after downloading. As reported previously [43], interest in using the app was associated both with perceived benefits and with perceptions that someone at the clinic would assist them on how to use it. Although a detailed description of our study participants (both users and nonusers) is available elsewhere [43], we did not collect information on nonparticipants, who may have not participated because the study was not available in Spanish, they did not have the correct type of phone, or may have been concerned about costs for SMS services. Given the sample size of both app *users* and *nonusers*, we were limited in our ability to conduct subgroup analyses at this time. These would be valuable to further contextualize the app’s utility in subpopulations of PLWH, and future research on commercially available medication adherence apps should consider assessing these differences. We noticed that almost 50% of participants had user fatigue throughout this 90-day pilot study. Active updates and app maintenance and a more robust contingency system (such as gamification) might help to sustain novelty and address this concern. ART adherence data relied on self-report. Future research should include not only a larger sample and experimental design, but also an objective measure of medication adherence.

In summary, Care4Today Mobile Health Manager, a commercially available, biprogrammatic medication adherence app that operates with both iOS and Android operating systems, as well as SMS, was found feasible and acceptable for improving ART adherence among PLWH during a 3-month longitudinal study. Further clinical research needs to address user fatigue to improve platform usage.

## Appendix

Multimedia Appendix 1 Summary of responses to open-ended questions regarding the acceptability of the mobile health platform, Florida Mobile Health Adherence Project for People Living With HIV Study, 2016.

## Abbreviations

ART: antiretroviral therapy
FL-mAPP: Florida mHealth Application Adherence Project
mHealth: mobile health
PLWH: people living with HIV
