# Supplementary material for: The Florida Mobile Health Adherence Project for People Living With HIV (FL-mAPP): Longitudinal Assessment of Feasibility, Acceptability, and Clinical Outcomes
Source: JMIR Mhealth Uhealth. 2020 Jan 8;8(1):e14557. doi: 10.2196/14557 (PMC6996722; doi:10.2196/14557)
Supplement: Multimedia Appendix 1 [file mhealth_v8i1e14557_app1.docx]

| **Questions and key words** | | **Responses** |
| --- | --- | --- |
| What stopped you from using the Care4Today platform more? | | |
| *Forgetfulness* | -“Just forgot to use it.”  -“I forget about it, but the app is helping with that.” | |
| *Technical issues* | -“It would not send alerts, so I had to click on the app every day to use it.”  -“Patient already used it daily. Sometimes gets locked out of the app.”  -“Had a hard time setting reminders for other meds.”  -“Phone got broken, and then got locked out.”  -“Alarm didn’t go off for a week straight and so I forgot about app—and had to go back in to reconfigure.” | |
| What did you not like about the Care4Today platform? | | |
| *Features* | -“Had trouble because I had trouble signing in. Small keypad and I have big fingers.”  -“Can’t enter if medication was taken later than recommended time.”  -“I was expecting on receiving some type of sound (beep, chime, etc.) as a daily reminder.”  -“I can’t let it know I’ve taken my meds early. Even if it’s a small amount of time. I just have to remember when I wake to let it know.” | |
| *Technical issues* | -“It would not send me reminders unless I went into the app myself.”  -“Doesn’t work without wifi.”  -“It didn’t know my medication readily in the system of pre-registered meds.”  -“It just stopped working out of nowhere.”  -“Sometimes rings late. Doesn’t keep going off until you take it. Doesn’t ring if your phone on silent/vibrate.”  -“I have data restriction plan. It wouldn’t work without data plan.” | |
| How can the Care4Today platform be improved? | | |
| *Features* | -“Make sure it sends text every day, not sometimes.”  -“The refill reminder can be simpler.”  -“Consistent alarm instead of just popping up in status bar to call more attention to it. It needs to keep ringing until I turn it off when I take my meds. More often than not it’s late sending the alarm.”  -“Instead of a chime that I often don’t hear, a more aggressive alarm that I have to turn off would work better for me.” | |
| *Technical features* | -“A little faster loading.” | |
| *User participation* | -“Continued research in involving actual app user will aid in improvement the ‘human’ connection.” | |
